# Metabolism of Imidazole Dipeptides, Taurine, Branched-Chain Amino Acids, and Polyamines of the Breast Muscle Are Affected by Post-Hatch Development in Chickens

**DOI:** 10.3390/metabo12010086

**Published:** 2022-01-17

**Authors:** Shozo Tomonaga, Takahiro Kawase, Takamitsu Tsukahara, Yoshiyuki Ohta, Jun-ichi Shiraishi

**Affiliations:** 1 Graduate School of Agriculture, Kyoto University, Kyoto 606-8502, Japan; 2 Kyoto Institute of Nutrition & Pathology, Ujidawara-cho, Tsuzuki-gun, Kyoto 610-0231, Japan; kawase@kyoto-inp.co.jp (T.K.); tsukahara@kyoto-inp.co.jp (T.T.); 3 Department of Animal Science, Nippon Veterinary and Life Science University, Tokyo 180-8602, Japan; ohta-y@nvlu.ac.jp

**Keywords:** metabolomics, skeletal muscle, plasma, growth, carnosine, insulin/IGF-1 signaling

## Abstract

To explore metabolic characteristics during the post-hatch developmental period, metabolomic analyses of breast muscle and plasma were performed in chickens. The most significant growth-related changes in metabolite levels were observed between seven and 28 days of age. Some of these metabolites are essential nutrients or reported as growth-promoting metabolites. In the muscle, two imidazole dipeptides—carnosine and its methylated metabolite, anserine—increased with the development. These dipeptide levels may be, in part, regulated transcriptionally because in the muscle mRNA levels of carnosine synthase and carnosine methylation enzyme increased. In contrast, taurine levels in the muscle decreased. This would be substrate availability-dependent because some upstream metabolites decreased in the muscle or plasma. In branched-chain amino acid metabolism, valine, leucine, and isoleucine decreased in the muscle, while some of their downstream metabolites decreased in the plasma. The polyamines, putrescine and spermidine, decreased in the muscle. Furthermore, mRNA levels associated with insulin/insulin-like growth factor 1 signaling, which play important roles in muscle growth, increased in the muscle. These results indicate that some metabolic pathways would be important to clarify metabolic characteristics and/or growth of breast muscle during the post-hatch developmental period in chickens.

## 1. Introduction

During the developmental period, remarkable increases in body and tissue weights are observed in animals. In general, diet quality is an important factor for the development. In the diet, growth-promoting effects may not only be induced by essential nutrients such as essential amino acids and vitamins, but also by bioactive metabolites. In chickens, these dietary metabolites have been extensively investigated because improvement of the growth performance is important for the poultry industry. Some studies on them are shown in detail from the next paragraph [1,2,3,4,5,6,7,8,9].

Dietary carnosine, an imidazole dipeptide, can positively affect the growth of chickens in some cases. Dietary carnosine (0.01, 0.02, or 0.04%) administered for 21 days (from one to 21 days of age) improved the feed-to-gain ratio, while body weight gain was not affected [1]. The same effects were observed with dietary 0.04% carnosine for 28 days (from 72 to 100 days of age) [2]. In addition, the effects of dietary β-alanine, which is one of the constituent amino acids of imidazole dipeptides (carnosine, anserine, and balenine), on growth performance was confirmed. Dietary β-alanine (0.25, 0.5, 1, or 2%) administered for 21 days (from one to 21 days of age) increased the body weight gain and breast muscle yield and improved the feed-to-gain ratio [3]. In contrast, dietary β-alanine (1% or 2%) fed for 28 days (from 28 to 56 days of age) decreased the body weight gain and breast muscle weight [4]. These reports suggest that the positive effects of imidazole dipeptide metabolism on growth performance in chickens could be observed under certain experimental conditions, although the underlying mechanisms are still not clear.

Taurine, which shares a transporter with β-alanine [10], is one of the downstream metabolites of sulfur amino acids and can also positively affect growth in chickens. Dietary taurine (0.25%, 0.5%, or 0.75%) fed for 42 days (from one to 42 days of age) increased body weight gain and improved the gain-to-feed ratio in chickens [5]. In addition, 0.5% dietary taurine administered for 14 days (from 28 to 42 days of age) has been reported to ameliorate the decrease in breast muscle weight induced by chronic heat stress without affecting the heat stress-induced negative effects on body weight gain and feed-to-gain ratio in chickens [6]. However, the detailed mechanisms of these effects on growth performance have not yet been clarified.

In some other amino acid metabolic pathways, certain metabolites have been reported to affect growth in chickens. The leucine metabolite 3-hydroxy-3-methylbutyric acid (HMB) can improve growth performance. Dietary HMB-Ca (0.1 or 0.5%) administered for 42 days (from one to 42 days of age) increased body weight gain and decreased mortality in a dose-dependent manner [7]. The treatment dose-dependently increased breast muscle yield and decreased abdominal fat [7]. In addition, two polyamines (putrescine and its downstream metabolite spermine), which are metabolites of arginine, have been reported to affect growth. Dietary 0.2% putrescine administered for 14 days (from seven to 21 days of age) increased body weight gain, while a higher dose (0.8 or 1.0%) decreased the gain [8]. In contrast, dietary spermine (0.2%, 0.8%, or 1.0%) decreased body weight gain [9]. The mechanisms of the action of HMB or polyamines on growth performance have not yet been clarified.

From the hormonal point of view, insulin and insulin-like growth factor 1 (IGF-1) have been known as important factors for post-hatch nutrition and growth [11]. Among the signaling pathways of these hormones, insulin receptor substrate 1 and insulin receptor substrate 2 are major substrates for both insulin and IGF-I receptors; they are suggested to be involved in growth in rodents [12,13]. In chicken myotube cultures, both insulin and IGF-1 can stimulate muscle growth [14,15]. However, the relationships among post-hatch growth, insulin/IGF-1 signaling, and dietary supplementation of bioactive metabolites have not yet been understood clearly in animals, including chickens.

Thus, growth-promoting metabolites should be investigated in more detail. For this purpose, we speculate that a comprehensive investigation of metabolite levels during the post-hatch developmental period in chickens will be useful. For example, if developmental changes of growth-promoting metabolite levels are linked to the physiology of growth, it could be important information to determine better supplemental dose and/or period of the metabolites. Therefore, in the present study, metabolomic analyses of plasma and breast muscle were performed in chickens during the post-hatch developmental period (7, 28, and 42 days of age; P7, P8, and P42, respectively). In addition, the mRNA expression of metabolic enzymes and factors in insulin/IGF-1 signaling were also investigated to explore the molecular mechanisms linked to growth.

## 2. Results

### 2.1. Body and Tissue Weights

Body and tissue weights were clearly higher with age (Table 1). These results suggest that the present experimental conditions were, to some extent, suitable for focusing on post-hatch growth in chickens.

### 2.2. Targeted and Non-Targeted Metabolomic Analysis

#### 2.2.1. Quantitative Analyses of Imidazole Dipeptides and Taurine in Breast Muscle and Plasma

In the breast muscle, among the three imidazole dipeptides, carnosine and anserine levels increased significantly with the development, while balenine level did not change (Table S1). As a result, the total imidazole dipeptide levels increased (Table S1). In contrast, the taurine level decreased (Table S1). Significant changes were detected between P7 and P28, but not between P28 and P42.

In the plasma, carnosine, anserine, total imidazole dipeptides, and taurine levels did not change with the development (Table S1).

#### 2.2.2. Non-Targeted and Semi-Quantified Analyses of Metabolites in the Breast Muscle and Plasma

Of the 103 metabolites semi-quantified in the breast muscle (Tables S2–S4), 48 showed decreased levels with the development (Table S2), while the levels of three metabolites increased (Table S3). In addition, of the 61 metabolites that were significantly affected, the levels of 43 metabolites changed significantly between P7 and P28, while only one metabolite (mesaconic acid) changed significantly between P28 and P42.

Of the 125 metabolites semi-quantified in the plasma (Tables S5–S7), the levels of 21 decreased with the development (Table S5), and those of 18 metabolites increased (Table S6). In addition, of the 50 metabolites that were significantly affected, the levels of 31 metabolites changed significantly between P7 and P28, while five metabolites changed significantly between P28 and P42.

#### 2.2.3. Pathway Analyses in the Breast Muscle and Plasma

Data from the aforementioned analyses (Tables S1–S7) were integrated and analyzed using pathway analysis. Analyses of the pathways associated with the significantly affected metabolites revealed that 16 pathways in the muscle and nine pathways in the plasma were potentially affected by post-hatch development (Table 2). A metabolic pathway number (Metab. No.) was assigned to each pathway (Table 2). Metab. Nos. 1–3 are common pathways suggested in the breast muscle and plasma. Metab. Nos. 4–16 are pathways suggested only in breast muscle. Metab. Nos. 17–22 are the pathways suggested only in the plasma.

#### 2.2.4. Focusing on the Metabolites Based on the Pathway Analyses

Based on the pathway analyses (Table 2), metabolites were selected and divided into four groups (Table 3, Table 4, Table 5 and Table 6).

Metab. Nos. 7 and 12 are carnosine-related metabolic pathways; they are only significantly found in the breast muscle. The related metabolite levels in the breast muscle and plasma are listed in Table 3. In addition to the effects on imidazole dipeptides described in Section 2.2.1, spermidine decreased with the development in the muscle.

Metab. Nos. 8 and 16 are metabolic pathways of sulfur-containing amino acids, including taurine; they are only significantly found in the breast muscle. The related metabolite levels in the breast muscle and plasma are listed in Table 4. In addition to the effects on taurine itself, the effects of the development on the upstream metabolites of taurine were clearly observed. Homocystine levels decreased in the muscle and plasma. Cystathionine, homocysteine, hypotaurine, and methionine levels decreased in the muscle.

Metab. Nos. 1 and 22 are metabolic pathways of branched-chain amino acids (BCAAs); they are significantly found in the breast muscle and/or plasma. The related metabolite levels in the breast muscle and plasma are listed in Table 5. Valine, leucine, isoleucine, and glutamic acid, which could be downstream metabolites of them, decreased in the muscle. BCAA metabolites (2-hydroxyisovaleric acid, 2-hydroxy-3-methylvaleric acid, 2-keto-isovaleric acid, 2-ketoisocaproic acid, 2-methyl-3-hydroxybutyric acid, 3-methyl-2-oxovaleric acid, and HMB) decreased in the plasma.

Metab. No. 5 is a metabolic pathway of polyamines and related amino acids; it is found in the breast muscle. The related metabolites are listed in Table 6. Polyamines (putrescine and spermidine) and related amino acids (arginine, ornithine, proline, and glutamic acid) were decreased in the muscle.

#### 2.2.5. mRNA Levels of Metabolic Enzymes and Insulin/IGF-1 Signaling-Related Factors

**Figure 1:**
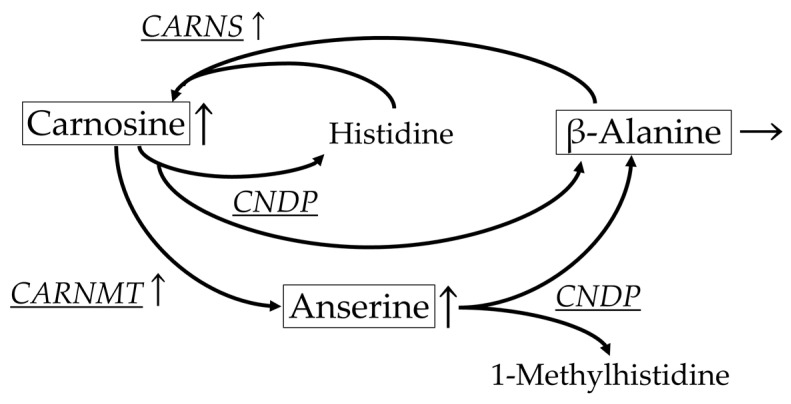
Carnosine-related metabolism during post-hatch developmental period. Boxed metabolites are quantified or semi-quantified metabolites of the breast muscle during the development in chickens. Metabolic enzymes were shown with underbar. Arrows indicate increase (↑) or no effect (→) for each metabolite level or metabolic enzyme mRNA level.

Based on the obtained results regarding the levels of different metabolites (Table 3, Table 4, Table 5 and Table 6), we selected related metabolic enzymes, and measured their mRNA levels (Table 7). An increase or decrease was observed in the mRNA levels of six metabolic enzymes. Among the mRNA levels, an increase was observed in carnosine synthesis (*CARNS*) and carnosine methylation enzyme (*CARNMT*), and a tendency to decrease was observed in imidazole dipeptide degradation enzyme (*CNDP*). These changes seem to be linked to the corresponding metabolite levels (Table 3). Therefore, we created a figure to explain these changes (Figure 1).

Because insulin/IGF-1 signaling contributes to growth, as mentioned in the introduction, we checked the mRNA levels of the signaling-associated factors (Table 7). Increases with the development were observed at all the mRNA levels.

## 3. Discussion

Considering the body and tissue weights shown in Table 1, the sampling time points in the present study (P7, P28, and P42) clearly included the growth stage. In addition, it is obvious that growth was more rapid between P7 and P28 than between P28 and P42. Among the metabolites, significant changes were detected, most of which were between P7 and P28. Therefore, in the present experimental conditions, we could confirm metabolic changes during the post-hatch developmental period, especially between P7 and P28. Future studies that focus on the period where chickens are younger than the P28 stage and use more sampling time points will explain the metabolic changes during post-hatch developmental in more detail.

Pathway analyses suggested that between the breast muscle and plasma, some metabolic changes during the post-hatch developmental stage may be similar, while others are in contrast. These results suggest that the development may affect metabolism differently in the muscle and plasma. Of all the suggested metabolic pathways, those for certain growth-promoting metabolites were confirmed. Therefore, they are discussed in the next section.

In the breast muscle, the levels of carnosine and its methylated metabolite, anserine increased with the development. These dipeptide levels during this period may be, to some extent, regulated transcriptionally. This is because mRNA levels of carnosine synthase and carnosine methylation enzyme increased in the muscle while that of the degradation enzyme tended to decrease. However, plasma carnosine and anserine levels did not change with the development. These results suggest that carnosine and anserine levels in the plasma did not reflect the degree of post-hatch development, but their levels in the breast muscle did. According to a previous study, dietary β-alanine not only improved body weight gain, breast muscle yield, and feed-to-gain ratio, but it also increased β-alanine and carnosine levels (but not anserine level) in the breast muscle [3]. According to another study, which was conducted under the experimental conditions that caused dietary β-alanine to induce negative effects on growth, β-alanine levels increased while carnosine and anserine levels did not change in the breast muscle [4]. In addition, two studies suggested that dietary carnosine improved the feed-to-gain ratio; however, they did not confirm the β-alanine, carnosine, and anserine levels in breast muscles [1,2]. Therefore, it is important to investigate the relationship between the metabolism of imidazole dipeptides in the muscle and growth in chickens in more detail.

In contrast to carnosine and anserine levels in the breast muscle, taurine level in the muscle decreased with the development. This might be attributed to substrate availability because the levels of some upstream metabolites, such as cystathionine, homocysteine, homocystine, hypotaurine, and methionine, decreased in the muscle or plasma. Of note is that among these metabolites, methionine is an essential amino acid. This decrease was confirmed in the muscle but not in the plasma. Therefore, the present results may reflect changes in methionine requirements in the breast muscle during post-hatch development. Focusing on taurine itself, plasma taurine level did not change with the development. Therefore, taurine levels in the plasma did not reflect the degree of post-hatch growth, and its level in the breast muscle during this period did. Previous studies have not evaluated the growth-promoting effects of dietary taurine, focusing on levels of taurine and its upstream metabolites in chickens [5,6]. Considering the present results, it may be useful to evaluate whether the growth-promoting effects of dietary taurine are linked to growth-related changes in taurine metabolism in the muscle.

In BCAA metabolism, valine, leucine, isoleucine, and glutamic acid (a downstream metabolite of valine, leucine, and isoleucine) decreased in the muscle. Some BCAA metabolites decreased in the plasma while valine, leucine, and isoleucine did not. These results could be linked to the transcription of their metabolic enzymes because the mRNA levels of these enzymes in the breast muscle changed. Importantly, BCAAs are essential amino acids in chickens, and therefore, the present results may reflect the requirement of these essential nutrients in the muscle. Focusing on other samples, such as other muscles, liver, fat, and cecal content, will be important to understand comprehensively. In BCAA metabolites, HMB in the plasma decreased with the development. Dietary HMB-Ca has been reported to have a growth-promoting effect, while the HMB levels in tissues and blood have not been measured [7]. To elucidate the mechanism of the dietary effect of HMB, it may be important to take into consideration the developmental changes of HMB levels in tissues and blood.

The levels of polyamines, putrescine and spermidine, were decreased in the muscle. Polyamine metabolism in the muscle may be inactivated in a substrate-dependent manner with the development because its upstream metabolites, arginine and ornithine, also decreased in the muscle. No changes were observed in the mRNA levels for the enzymes for polyamine synthesis in the muscle. This suggested that the transcription of these enzymes in the muscle was not related to the polyamine and its upstream metabolite levels. Previous reports suggest that dietary putrescine has a growth-promoting effect [8], while spermine, a downstream metabolite of spermidine, induces growth retardation [9]. However, these reports did not evaluate the effects of these metabolites on breast muscle weight and polyamine-related metabolite levels in the muscle. To clarify the effects of polyamines on growth, it may be valuable to clarify polyamine metabolism in the breast muscle during the development in more detail.

The mRNA levels of factors associated with insulin/IGF-1 signaling increased in the breast muscle with the development. This may be attributed to the activation and/or sensitivity of receptors and/or downstream signaling pathways. The signaling plays an important role in the muscle growth in chickens [14,15]. Furthermore, it should be noted that in other animal species, the two metabolites whose levels got altered in the present study with the development can stimulate insulin or IGF-1 secretion. Oral administration of leucine stimulates insulin secretion in humans [16]. In addition, intake of taurine through rat milk during the first few days after birth stimulates the growth of baby rats by increasing the secretion of IGF-1 [17]. Therefore, it is possible that the levels of these metabolites changed with the development, contributing to insulin/IGF-1 signaling in chickens. Further studies should focus on the relationships among insulin/IGF-1 signaling, post-hatch growth, and metabolites altered with the development, especially leucine and taurine.

Taken together, the present study suggests that some amino acid metabolic pathways could be related to metabolic characteristics and/or growth of breast muscle during post-hatch developmental period in chickens. For a more comprehensive understanding, more metabolic information linked to growth and biological properties of related metabolites will be needed. Focusing on other samples such as other muscles, liver, fat, and cecal content will be effective. In addition, comparison between strains with different growth rates will be useful. One of the limitations of the present study is that only certain amino acid metabolic pathways linked to some bioactive metabolites were focused on. Therefore, other metabolic pathways which were not focused on but suggested in the present study may be worth investigating. Further studies using metabolomics will be effective and should be conducted in the future.

## 4. Materials and Methods

### 4.1. Animals

Fertilized eggs were purchased from Single Comb White Leghorn (Japan Layer, Gifu, Japan) and incubated at a temperature of 37.8 °C and a relative humidity of 60%. Hatched male chicks were reared in electronic heating cages (174 × 60 × 30 cm, floor space; 1.044 m^2^) under group housing conditions and given free access to a commercial standard diet (JA Higashinihon Kumiai Shiryou Co. Ltd., Gunma, Japan, Metabolic Energy 2.9 M cal/kg, Crude Protein 22%, Crude Fat 3.5%, Crude Fiber 5.0%, Crude Ash 8.0%, Calcium 0.90%, Phosphorus 0.6%) and water until the experiment ended. The chicks were bred under 24 h lighting in a cage maintained at an environmental temperature of 32 °C for up to 28 d, after which the chicks were bred in a floor rearing under controlled conditions at 24 °C. Fifteen hatched chicken male chicks were bred, and then 5 birds were sampled at 7, 28, and 42 days after hatching. On the day of the experiment (P7, P28, and P42), the birds were sacrificed by decapitation after body weight measurement and blood collection from the wing vein. The left breast muscle was then collected and weighed. Then, a tissue sample with dimensions of about 1 cm square was collected from the center of the muscle, snap frozen in liquid nitrogen, and stored at −85 °C until assayed. Harvest plasma was stored at −85 °C until plasma metabolite analysis. This animal experiment was conducted over 6 weeks at the attached experimental facility of Nippon Veterinary and Life Science University (Musashino City, Tokyo). The university facility is located at an altitude of 60 m at 35°42′ N and 139°32′ E.

### 4.2. Sample Preparation for Gas Chromatography/Mass Spectrometry Analysis

Metabolomic analysis was performed using gas chromatography/mass spectrometry (GC/MS) as previously described [18], with some modifications. Approximately 20 mg of the freeze-dried muscle samples or 50 μL of plasma was suspended in 250 µL of methanol–chloroform–water (5:2:2) and 5 µL of 1 mg/mL 2-isopropylmalic acid as an internal standard. The samples were then mixed in a shaker at 1200 rpm at 37 °C for 30 min and then centrifuged at 16,000× *g* at 4 °C for 5 min. Next, 225 µL of the supernatant was mixed with 200 µL of distilled water and vortexed, followed by centrifugation at 16,000× *g* at 4 °C for 5 min. Subsequently, 250 µL of the supernatant was dried under vacuum using a centrifugal evaporator (RD-400; Yamato Scientific, Tokyo, Japan) after cooling at −80 °C for 10 min. Methoxyamine hydrochloride in pyridine (20 mg/mL, 40 µL) was then added to the tubes, vortex-mixed, and shaken at 1200× *g* at 30 °C for 90 min in the dark for oximation. *N*-methyl-*N*-trimethylsilyltrifluoroacetamine (20 µL) was then added to each tube, and the contents were vortex-mixed. Trimethylsilyl derivatives were prepared by shaking the tubes at 1200× *g* at 37 °C for 45 min in the dark.

### 4.3. GC/MS Analysis

GC/MS analysis was performed using a GCMS-QP2010 Ultra (Shimadzu, Kyoto, Japan) according to a previously described method with some modifications [18]. A DB-5 column (30 m × 0.25 mm, internal diameter (i.d.); film thickness 1.00 μm; Agilent, Tokyo, Japan) was used for GC. The GC column temperature was programmed to maintain an initial temperature of 100 °C for 4 min, then increased to 320 °C at a rate of 10 °C/min, and finally to remain at 320 °C for 11 min, producing a total GC run time of 37 min. The inlet temperature was maintained at 280 °C, and helium was used as the carrier gas at a constant flow rate of 39.0 cm/s. A sample of 1.0 μL was injected in splitless mode, and the mass conditions were as follows: Ionization voltage, 70 eV; ion source temperature, 200 °C; full scan mode in the m/z range 45–600 at an interval of 0.3 s. The chromatogram acquisition and detection of mass spectral peaks and the processing of their waveforms were performed using Shimadzu GC/MS solution software (Shimadzu, Kyoto, Japan).

### 4.4. Data Processing for GC/MS Data

The GC/MS analysis data were exported in net CDF format, converted to ABF format, and peak detection and alignment were performed using MS-DIAL version 2.76 (RIKEN Center for Sustainable Resource Science, Yokohama, Kanagawa, Japan) [19]. To minimize the number of missing values, peaks with a similarity of >70% and a retention index within ±10% were accepted by comparison with the Smart Metabolites Database (Shimadzu, Kyoto, Japan). Metabolite levels were semi-quantified using the peak area of each metabolite relative to the internal standard (2-isopropylmalic acid). The mean of each metabolite in the P7 chickens was set to 100.

Sample preparation for ultra-performance liquid chromatography-tandem mass spectrometry

For the standard substances, carnosine, anserine, and taurine were purchased from FUJIFILM Wako Pure Chemical (Osaka, Japan). Balenine was purchased from Hamari Chemicals (Osaka, Japan). A mixed standard solution was prepared in purified water to a concentration of 10–500 μmol/L. The internal standards were _L_-histidine:HCl:H_2_O (ring-2-^13^C) (Cambridge Isotope Laboratories, Tewksbury, MA, USA) and *L*-glutamine-2,3,3,4,4-d_5_ (C/D/N Isotopes, Pointe-Claire, QC, Canada). A mixed internal standard solution was prepared in purified water to 0.1 mg/mL, respectively. The AccQ-Tag Ultra Chemistry Kit (AccQ-Tag Ultra Column, AccQ-Tag Ultra borate buffer, AccQ-Tag Ultra reagent powder, and AccQ-Tag Ultra reagent diluent, AccQ-Tag Ultra eluents for ultra-performance liquid chromatography-tandem mass spectrometry (UPLC-MS/MS) analysis) was purchased from Waters (Milford, MA, USA). Three milligrams of AccQ-Tag Ultra reagent powder were eluted with 1 mL AccQ-Tag Ultra reagent diluent for 15 min at 55 °C and used as a derivatization reagent. The methods of sample preparation for measuring imidazole dipeptides and taurine were as described previously [20] with slight modifications. Briefly, 500 μL plasma samples were ultrafiltered using AmiconR Ultra-0.5 Centrifugal Filter Devices 30 K (Millipore, MA, USA) at 14,000× *g* for 15 min at 4 °C. Next, 20 μL filtrate was added to 10 μL mixed internal standard solution, 140 μL AccQ-Tag Ultra borate buffer, and 40 μL derivatization regent and vortex-mixed. After incubation for 15 min at 55 °C, the solution was cooled and transferred to glass vials for analysis. Approximately 100 mg of freeze-dried breast muscle samples were dispensed into 2 mL screw-cap tubes (Watson, Tokyo, Japan), and 400 μL 0.2 mol/L perchloric acid (FUJIFILM Wako Pure Chemical, Osaka, Japan) and a φ 5.5 stainless steel ball (TOMY SEIKO, Tokyo, Japan). The samples were homogenized at 3000 rpm for 30 s using a Micro Smash MS-100 (TOMY SEIKO), and left to stand on ice for 30 min in the dark for deproteinization. After centrifugation at 15,000× *g* at 4 °C for 15 min, the supernatant was filtrated by 0.2 μm syringe filter (mdi Membrane Technologies, PA, USA). Next, 20 μL of the filtrate was added to 5 μL mixed internal standard, 140 μL AccQ-Tag Ultra borate buffer, 40 μL derivatization reagent, and vortex-mixed. After incubation for 15 min at 55 °C, the solution was cooled and transferred to glass vials for analysis.

### 4.5. UPLC-MS/MS Analysis

The UPLC-MS/MS conditions were adjusted as previously described [20], with some modifications. Briefly, a UPLC device equipped with a binary solvent manager, an autosampler, a column heater connected to an AccQ-Tag Ultra column (2.1 i.d. × 100 mm, 1.7 μm particles), and Acquity TQD tandem mass spectrometry (Waters) was used to detect the imidazole dipeptides and taurine. The column heater was set at 55 °C, and the mobile phase was 10-fold diluted AccQ-Tag Ultra eluent A using purified water as solution A and AccQ-Tag Ultra eluent B as solution B. The gradient procedure was as follows: Solution A was 99.9% (0–0.54 min), 99.9–90.9% (0.54–5.74 min), 90.9–78.8% (5.74–7.74 min), 78.8–40.4% (7.74–8.04 min), 40.4–10% (8.04–8.05 min), 40.4% (8.05–8.64 min), 10–99.9% (8.64–8.73 min), 99.9% (8.73–9.5 min). The flow rate was set at 0.7 mL/min, and the gradient curves were linear curves, except for 0.54–5.74 min, which was a concave curve (curve number 7 of Waters original parameters). The injection volume was 1 µL. Ionization and detection were carried out using a Waters TQD tandem quadrupole detector with a Z-spray ion interface in positive electrospray ionization mode. The source temperature was set at 150 °C. The capillary voltage was set at 3.0 kV. Nitrogen was used as the desolvation gas at 450 °C under a 900 L/h flow rate. Multiple reaction monitoring parameters, including cone voltage and collision energy, are shown in Table S8. Metabolite levels were quantified using the peak area of each metabolite and each internal standard. For the calculation of relative values, the mean of each metabolite in the P7 chickens was set to 100.

### 4.6. Quantification of mRNA Levels by Real-Time PCR

RNA was isolated from the dissected tissue using RNAiso Plus (Takara, Shiga, Japan) according to the manufacturer’s instructions. To rule out the possibility that the PCR products would result from the amplification of genomic DNA contaminating the RNA sample, RNA samples were treated with DNase I using a DNA-free kit (Thermo Fisher Scientific, Massachusetts, USA). Total RNA (350 ng) was reverse transcribed at 37 °C for 15 min in 10 μL of 1 × PrimeScript buffer containing 50 μM random primers and Prime Script RT Enzyme Mix I (Takara, Shiga, Japan). The reaction product was subjected to qPCR according to the manufacturer’s instructions for the Applied Biosystems 7500 Fast Real-Time PCR System (Thermo Fisher Scientific, Massachusetts, USA). Briefly, following a denaturation step at 95 °C for 30 s, PCR was carried out with a thermal protocol consisting of 95 °C for 5 s and 60 °C for 34 s in 20 μL buffer containing 1 × SYBR Premix EX Taq (Takara, Shiga, Japan) and 0.4 μM of each primer. Ribosomal protein S17 (*RPS17*) was used as an endogenous control. The primer information used in this experiment is listed in Table S9. To normalize the data, ∆ C_T_ was calculated for each sample by subtracting the C_T_ of *RPS17* from the C_T_ of the gene of interest. For relative quantitation, ∆ C_T_ was the defined control group and was then subtracted from the ∆ C_T_ of each experimental sample to generate ∆∆ C_T_. ∆∆ C_T_ was then used to calculate the approximate fold difference, 2^∆∆CT^. The results were expressed as the gene of interest mRNA/*RPS17* mRNA ratio.

### 4.7. Statistical Analyses

At first, we checked all data by Shapiro–Wilk test for normality and Levene’s test for homogeneity of variances. Some of them violated normality and/or violated homogeneity of variances (data not shown). Therefore, we decided to use non-parametric analyses for all data to evaluate equally. Data comparisons were performed using Kruskal–Wallis test with Steel–Dwass post-hoc multiple comparison test. The JMP Pro software version 16.1.0 (SAS Institute, Cary, NC, USA) was used to perform these analyses. Metabolites significantly affected were subjected to pathway analysis using MetaboAnalyst 5.0 (https://www.metaboanalyst.ca/ accessed on 30 November 2021) [21]. *Gallus gallus* (Kyoto Encyclopedia of Genes and Genomes) was used for the pathway library. In these analyses, *p* < 0.05 was considered statistically significant.

## Figures and Tables

**Table 1:** Body and tissue weights during post-hatch developmental period in chickens.

	P7				P28				P42				KW *p*-Value
		Q1	Q2	Q3		Q1	Q2	Q3		Q1	Q2	Q3	
Initial body weight (g)		63.4	67.8	83.9		66.2	67.2	70.0		65.1	69.6	73.7	0.756
Final body weight (g)	C	63.4	67.8	83.9	B	335.3	337.0	341.8	A	605.0	640.0	660.0	<0.005
Breast muscle weight (g)	C	0.98	1.15	1.35	B	2.02	2.08	2.13	A	2.46	2.50	2.59	<0.005
Liver weight (g)	C	2.62	2.91	3.03	B	10.38	11.20	12.03	A	19.39	20.67	21.95	<0.005
Brain weight (g)	C	1.14	1.15	1.18	B	8.65	9.25	10.30	A	14.66	15.46	16.76	<0.005

P7, P28, and P42; 7, 28, and 42 days of age, respectively. Q1, Q2, and Q3; lower quartile, median quartile, and upper quartile, respectively. KW, Kruskal–Wallis test; Different letters in the same line denote significantly different according to Steel–Dwass test (*p* < 0.05). *n* = 5 in each group.

**Table 2:** Potential metabolic pathways affected by post-hatch development in chickens.

Metab. No.	Pathway Name	Breast Muscle	Plasma
1	Valine, leucine, and isoleucine biosynthesis	*	**
2	Alanine, aspartate, and glutamate metabolism	*	*
3	Glyoxylate and dicarboxylate metabolism	*	*
4	Aminoacyl-tRNA biosynthesis	**	
5	Arginine and proline metabolism	*	
6	Arginine biosynthesis	*	
7	β-Alanine metabolism	*	
8	Cysteine and methionine metabolism	*	
9	_D_-Glutamine and _D_-glutamate metabolism	*	
10	Glutathione metabolism	*	
11	Glycine, serine, and threonine metabolism	*	
12	Histidine metabolism	*	
13	Nitrogen metabolism	*	
14	Phenylalanine metabolism	*	
15	Phenylalanine, tyrosine, and tryptophan biosynthesis	*	
16	Taurine and hypotaurine metabolism	*	
17	Ascorbate and aldarate metabolism		*
18	Citrate cycle		*
19	Glycerolipid metabolism		*
20	Pantothenate and CoA biosynthesis		*
21	Galactose metabolism		*
22	Valine, leucine, and isoleucine degradation		*

* *p* < 0.05; ** *p* < 0.0001. Metab. No. metabolic pathway number.

**Table 3:** Levels of imidazole dipeptides and related metabolites during post-hatch developmental period in chickens.

	P7				P28				P42				KW *p*-Value
		Q1	Q2	Q3		Q1	Q2	Q3		Q1	Q2	Q3	
**Breast muscle**													
Carnosine	B	76.6	104.8	121.0	A	188.9	222.7	308.1	A	205.9	280.2	354.4	<0.05
Anserine	B	71.9	95.0	130.6	A	212	311.7	376.4	A	232.2	273.7	338.5	<0.05
Balenine		84.9	100.6	114.8		140.8	154.1	175.5		57.5	158.2	180.2	0.055
Total imidazole dipeptides	B	76.8	91.2	127.6	A	204.7	318.7	337.4	A	242.1	291.4	315.5	<0.05
β-Alanine		58.3	104.7	139.3		98.7	121.8	169.7		63.8	98.8	148.7	0.472
3-Hydroxypropionic acid	A	78.3	107.8	117.8	A	109.3	134.3	160.0	A	127.5	173.3	187.9	<0.05
Aspartic acid		89.5	99.4	110.8		81.8	93.5	100.7		89.2	90.4	92.4	0.310
Uracil		84.9	102.6	113.8		92.6	113.9	126.3		93.1	100.9	103.3	0.527
Uridine		92.0	101.2	107.4		94.7	111.3	119.3		99.5	103.7	113.5	0.472
Spermidine	A	70.7	110.8	123.9	B	30.8	37.3	38.8	B	14.7	24.6	27.3	<0.05
**Plasma**													
Carnosine		45.5	85.5	161.7		41.3	61.9	91.6		83.1	118.8	259.3	0.168
Anserine		38.7	111.8	155.4		77.4	88.5	144.3		112.5	141.6	321	0.298
Total imidazole dipeptides		41.2	109.4	154.1		63.9	78.6	124.7		116.1	124.4	292.1	0.176
β-Alanine		85.7	99.8	114.4		95.6	102.2	131.1		94.7	117.4	151.5	0.432
3-Hydroxypropionic acid		91.9	100.1	108.0		97.0	101.5	103.6		89.9	93.5	106.2	0.403
Uracil		85.9	96.4	115.9		113.8	120.1	140.8		102.2	116.0	148.0	0.087
Uridine		83.9	105.2	113.5		108.5	112.8	158.9		98.1	109.2	200.3	0.310
Histidine		86.5	96.1	115.5		95.7	128	144.8		81.5	135.5	177.1	0.403

P7, P28, and P42; 7, 28, and 42 days of age, respectively. Q1, Q2, and Q3; lower quartile, median quartile, and upper quartile, respectively. KW, Kruskal–Wallis test; Different letters in the same line denote significantly different according to Steel–Dwass test (*p* < 0.05). The unit is a relative value. *n* = 4–5 in each group.

**Table 4:** Levels of taurine and related metabolites during post-hatch developmental period in chickens.

	P7				P28				P42				KW *p*-Value
		Q1	Q2	Q3		Q1	Q2	Q3		Q1	Q2	Q3	
**Breast muscle**													
Taurine	A	80.0	91.1	124.4	B	18.5	22.1	26.2	B	18.4	25.6	31.7	<0.05
Cystathionine	A	53.9	57.8	167.2	B	0.2	0.4	0.6	B	0.1	0.2	0.5	<0.05
Homocysteine	A	82.6	91.0	121.9	B	3.3	4.2	6.7	B	1.8	2.6	7.3	<0.05
Homocystine	A	53.9	69.7	161.3	B	1.2	3.6	9.7	B	4.7	8.1	11.5	<0.05
Hypotaurine	A	73.5	85.6	133.7	B	20.4	25.9	34.9	B	20.0	24.6	28.9	<0.05
Methionine	A	66.8	100.0	133.2	B	36.9	46.2	59.0	AB	39.1	49.6	64.1	<0.05
**Plasma**													
Taurine		22.0	76.5	189.8		25.6	43.0	131.3		123.5	181.7	198.2	0.131
Cystathionine		78.5	110.8	116.1		52.1	59.4	90.6		39.0	47.0	70.8	0.075
Cysteine		62.0	105.4	135.3		98.9	115.3	143.9		92.7	106.4	148.7	0.827
Cystine	A	86.5	100.8	113.1	A	38.2	55.1	75.9	A	60.2	94.6	103.3	<0.05
Homocysteine		69.0	110.2	125.9		68.6	79.5	122.8		51.0	68.6	110.5	0.566
Homocystine	A	74.4	109.5	120.8	B	15.5	27.2	31.5	C	4.9	6.3	7.9	<0.05
Hypotaurine		64.7	84.1	143.3		53.6	55.7	82.3		78.9	109.9	113.3	0.112
Methionine		77.7	97.7	123.4		77.8	86.7	99.8		95.8	117.8	138.6	0.141

P7, P28, and P42; 7, 28, and 42 days of age, respectively. Q1, Q2, and Q3; lower quartile, median quartile, and upper quartile, respectively. KW, Kruskal–Wallis test; Different letters in the same line denote significantly different according to Steel–Dwass test (*p* < 0.05). The unit is a relative value. *n* = 4–5 in each group.

**Table 5:** Levels of branched-chain amino acids and their metabolites during post-hatch developmental period in chickens.

	P7				P28				P42				KW *p*-Value
		Q1	Q2	Q3		Q1	Q2	Q3		Q1	Q2	Q3	
**Breast muscle**													
Valine	A	73.8	101.6	125.4	B	31.2	38.3	41.3	B	35.7	41.1	49.0	<0.05
Leucine	A	60.5	100.4	139.3	B	24.5	34.0	40.2	B	25.7	38.7	47.1	<0.05
Isoleucine	A	85.0	98.1	115.9	B	44.4	49.7	51.4	B	41.0	44.5	54.8	<0.05
Glutamic acid	A	83.2	103.6	115.0	B	29.1	32.3	40.7	B	29.8	34.4	39.0	<0.05
2-Keto-isovaleric acid		48.1	78.9	162.4		62.0	87.5	146.9		64.7	99.7	133.7	0.852
2-Methyl- 3-hydroxybutyric acid		11.2	118.6	179.5		27.7	126.0	255.4		48.9	72.0	112.1	0.651
**Plasma**													
Valine	A	79.4	110.0	115.6	A	48.8	54.2	77.3	A	64.3	81.2	83.0	<0.05
Leucine		83.3	103.5	114.9		56.9	62.1	85.5		75.3	88.3	94.4	0.061
Isoleucine		87.2	95.5	115.1		65.5	70.7	92.4		70.7	85.3	95.1	0.164
2-Hydroxyisovaleric acid	A	74.2	78.2	136.6	B	28.3	37.4	54.0	B	19.2	21.6	27.0	<0.05
2-Hydroxy- 3-methylvaleric acid	A	51.6	88.6	154.1	AB	20.0	41.4	62.6	B	8.7	9.9	14.3	<0.05
2-Keto-isovaleric acid	A	71.9	97.0	129.6	B	21.9	30.2	50.0	B	16.0	27.1	48.9	<0.05
2-Ketoisocaproic acid	A	86.0	96.6	115.7	AB	58.3	70.6	75.0	B	48.8	58.9	78.4	<0.05
2-Methyl- 3-hydroxybutyric acid	A	80.5	98.4	120.3	B	49.7	56.2	78.7	AB	52.7	68.7	86.6	<0.05
3-Methyl- 2-oxovaleric acid	A	82.8	94.8	119.8	B	41.5	54.7	65.0	B	34.8	47.0	61.5	<0.05
HMB	A	54.9	111.1	139.6	B	19.6	21.6	25.6	C	15.3	17.0	17.4	<0.05

P7, P28, and P42; 7, 28, and 42 days of age, respectively. Q1, Q2, and Q3; lower quartile, median quartile, and upper quartile, respectively. KW, Kruskal–Wallis test; Different letters in the same line denote significantly different according to Steel–Dwass test (*p* < 0.05). The unit is a relative value. *n* = 5 in each group. HMB: 3-hydroxy-3-methylbutyric acid.

**Table 6:** Levels of polyamines and related amino acids during post-hatch developmental period in chickens.

	P7				P28				P42				KW *p*-Value
		Q1	Q2	Q3		Q1	Q2	Q3		Q1	Q2	Q3	
**Breast muscle**													
Arginine	A	70.2	99.0	130.3	B	16.3	18.4	25.0	B	13.3	17.6	23.7	<0.05
Ornithine	A	75.7	94.0	127.3	B	17.8	21.2	27.0	B	15.2	20.2	24.2	<0.05
Proline	A	86.5	95.4	115.8	B	8.4	14.8	19.8	B	8.6	9.9	12.3	<0.05
Putrescine	A	66.7	94.3	136.1	B	18.9	22.6	28.0	B	11.3	15.5	17.8	<0.05
Spermidine	A	70.7	110.8	123.9	B	30.8	37.3	38.8	B	14.7	24.6	27.3	<0.05
Glutamic acid	A	83.2	103.6	115.0	B	29.1	32.3	40.7	B	29.8	34.4	39.0	<0.05
**Plasma**													
Ornithine		88.7	100.4	111.1		78.4	101.3	124.8		93.8	109.5	133.9	0.619
Proline		90.5	99.8	109.6		50.1	76.0	88.0		64.7	77.2	101.8	0.061

P7, P28, and P42; 7, 28, and 42 days of age, respectively. Q1, Q2, and Q3; lower quartile, median quartile, and upper quartile, respectively. KW, Kruskal–Wallis test; Different letters in the same line denote significantly different according to Steel–Dwass test (*p* < 0.05). The unit is a relative value. *n* = 5 in each group.

**Table 7:** Relative mRNA expressions of the breast muscle during post-hatch developmental period in chickens.

	P7				P28				P42				KW *p*-Value
		Q1	Q2	Q3		Q1	Q2	Q3		Q1	Q2	Q3	
Carnosine metabolism													
*CARNS*	B	79.1	93.7	124.0	AB	111.8	122.0	135.3	A	130.8	135.4	143.1	<0.05
*CNDP*	A	44.0	61.1	175.5	A	24.2	37.4	48.0	A	8.0	10.8	53.9	<0.05
*CARNMT*	B	83.4	101.5	115.8	A	141.9	148.0	150.7	A	142.9	170.0	180.8	<0.05
Sulfur amino acid degradation													
*CTH*	B	86.3	101.2	113.1	A	136.7	154.4	205.3	A	155.7	162.3	200.3	<0.05
Branched-chain amino acid degradation													
*BCAT*	A	86.1	93.2	117.3	A	79.4	83.8	103.7	B	47.1	49.4	60.1	<0.05
*BCKDH E1a*	A	87.7	94.4	115.1	A	120.8	141.0	156.0	A	109.1	116.4	139.7	<0.05
*BCKDH E1b*		84.3	92.9	119.2		68.4	82.4	98.4		75.3	78.3	95.4	0.265
Polyamine synthesis													
*ODC*		78.1	101.1	121.3		75.8	83.2	94.5		82.3	90.4	102.9	0.432
*AMD*		83.2	105.6	114.1		93.4	102.8	113.9		97.7	111.3	143.2	0.472
Insulin/IGF-1 signaling													
*INSR*	B	87.7	98.5	113.1	A	143.3	153.7	166.4	A	175.5	201.7	224.8	<0.05
*IGF1R*	B	83.9	96.7	117.7	AB	113.1	127.0	136.4	A	127.3	146.5	151.2	<0.05
*IRS1*	B	84.8	99.8	115.3	A	178.8	198.2	216.5	A	203.5	210.0	225.9	<0.05
*IRS2*	B	82.7	97.3	118.7	AB	116.0	145.5	182.7	A	157.2	184.4	256.7	<0.05

P7, P28, and P42; 7, 28, and 42 days of age, respectively. Q1, Q2, and Q3; lower quartile, median quartile, and upper quartile, respectively. KW, Kruskal–Wallis test; Different letters in the same line denote significantly different according to Steel–Dwass test (*p* < 0.05). The unit is a relative value. *n* = 5 in each group. Abbreviations: *AMD*, S-adenosylmethionine decarboxylase proenzyme; *CNDP1*, carnosine dipeptidase 1; *CARNMT*, carnosine N-methyltransferase; *CARNS*, carnosine synthase; *CTH*, cystathionine gamma-lyase; *BCKDH E1a*, branched-chain alpha-keto acid dehydrogenase E1-alpha subunit, *BCKDH E1b*: Branched-chain alpha-keto acid dehydrogenase E1-beta subunit; *INSR*, insulin receptor; *IGF1R*, insulin-like growth factor 1 receptor; *IRS1*, insulin receptor substrate 1; *IRS2*, insulin receptor substrate 2; *ODC*, ornithine decarboxylase.

## Data Availability

Data is contained within the article or Supplementary Materials.

## Supplementary Materials

The following supporting information can be downloaded at: https://www.mdpi.com/article/10.3390/metabo12010086/s1, Table S1: Metabolites quantified in the breast muscle and plasma, Table S2: Metabolites decreased in the breast muscle, Table S3: Metabolites increased in the breast muscle, Table S4: Other metabolites in the breast muscle, Table S5: Metabolites decreased in the plasma, Table S6: Metabolites increased in the plasma, Table S7: Other metabolites in the plasma, Table S8: LC-MS/MS conditions, Table S9: Primers for PCR. Data: all data in this study.

## Abbreviations

HMB: 3-hydroxy-3-methylbutyric acid; IGF-1: insulin and insulin-like growth factor; ANOVA: analysis of variance; Metab. No.: metabolic pathway number; BCAA: branched-chain amino acid; *AMD*: S-adenosylmethionine decarboxylase proenzyme; *CNDP1*: carnosine dipeptidase 1; *CARNMT*: carnosine N-methyltransferase; *CARNS*: carnosine synthase; *CTH*: cystathionine gamma-lyase; *BCKDH E1a*: branched-chain alpha-keto acid dehydrogenase E1-alpha subunit; *BCKDH E1beta*: branched-chain alpha-keto acid dehydrogenase E1-beta subunit; *INSR*: insulin receptor; *IGF1R*: insulin-like growth factor 1 receptor; *IRS1*: insulin receptor substrate 1; *IRS2*: insulin receptor substrate 2; *ODC*: ornithine decarboxylase.
