# Supplementary material for: Metabolism of Imidazole Dipeptides, Taurine, Branched-Chain Amino Acids, and Polyamines of the Breast Muscle Are Affected by Post-Hatch Development in Chickens
Source: Metabolites. 2022 Jan 17;12(1):86. doi: 10.3390/metabo12010086 (PMC8778354; doi:10.3390/metabo12010086)
Supplement: Supplementary file 1 [file metabolites-12-00086-s001.zip › Table S1 Metabolites quantified in the breast muscle and plasma.pdf]

Table S1. Metabolites quantified in the breast muscle and plasma of chickens

		P7				P28				P42			KW
		Q1	Q2	Q3		Q1	Q2	Q3		Q1	Q2	Q3	*p*-value
**Breast muscle**													
Carnosine*	B	4936	6753	7802	A	12178	14354	19862	A	13270	18060	22844	<0.05
Anserine*	A	10660	14081	19360	B	31423	46199	55795	B	34417	40569	50172	<0.05
Balenine*		94	111	127		156	171	194		64	175	200	0.054
Total imidazole dipeptides*	B	16420	19501	27281	A	43760	68139	72141	A	51762	62307	67453	<0.05
Taurine*	A	1355	1542	2106	B	313	374	443	B	311	433	537	<0.05
**Plasma**													
Carnosine**		1.14	2.15	4.07		1.04	1.56	2.31		2.09	2.99	6.52	0.168
Anserine**		1.64	4.74	6.59		3.28	3.75	6.12		4.77	6	13.61	0.298
Total imidazole dipeptides**		2.79	7.39	10.41		4.32	5.31	8.42		7.84	8.41	19.74	0.176
Taurine**		17.6	61.15	151.78		20.51	34.37	105		98.75	145.31	158.48	0.131

P7, P28, and P42; 7, 28, and 42 days of age, respectively. Q1, Q2, and Q3; lower quartile, median quartile, and upper quartile, respectively. KW, Kruskal-Wallis test; Different letters in the same line denote significantly different according to Steel-Dwass test ($p < 0.05$). *The unit in the breast muscle is nmol/g. **The unit in the plasma is μM. n=4-5 in each group.
