# Supplementary material for: Metabolism of Imidazole Dipeptides, Taurine, Branched-Chain Amino Acids, and Polyamines of the Breast Muscle Are Affected by Post-Hatch Development in Chickens
Source: Metabolites. 2022 Jan 17;12(1):86. doi: 10.3390/metabo12010086 (PMC8778354; doi:10.3390/metabo12010086)
Supplement: Supplementary file 1 [file metabolites-12-00086-s001.zip › Table S2 Metabolites decreased in the breast muscle.pdf]

Table S2. Metabolites decreased during postnatal development in the breast muscle of chickens

		P7				P28				P42			KW
		**Q1**	**Q2**	**Q3**		**Q1**	**Q2**	**Q3**		**Q1**	**Q2**	**Q3**	***p*-value**
3-Hydroxybutyric acid	A	78.8	93.4	124.5	AB	47.7	55.6	79.5	B	49.6	67.5	68.5	<0.05
Glyceric acid	A	64.4	101.1	135	AB	40.3	52.8	75.3	B	21.6	32.7	36.1	<0.05
Lysine	A	57.5	102.8	141.1	AB	24.3	43.5	58.3	B	14.5	18.9	26.6	<0.05
meso-Erythritol	A	86	101.9	113	AB	56.7	65.6	73.7	B	43.9	45.1	56.5	<0.05
Myristic acid	A	89.6	93.8	113.5	AB	73.1	75.8	98.7	B	60.9	70.9	75.6	<0.05
Tyrosine	A	76.3	107.8	119.8	AB	46.3	57.4	73.8	B	45.7	50.4	69.1	<0.05
Methionine	A	66.8	100.0	133.2	B	36.9	46.2	59.0	AB	39.1	49.6	64.1	<0.05
Pantothenic acid	A	88.8	99.0	111.7	B	47.4	60.6	69.9	AB	60.2	64.0	88.0	<0.05
Sorbose	A	35.4	89.6	169.8	B	14.9	20.5	26.7	AB	13.7	16.1	39.9	<0.05
15-Anhydro-glucitol	A	43.7	93.6	159.5	B	17.5	23.1	31.4	B	10	15.2	21.2	<0.05
2-Aminobutyric acid	A	74.5	91.9	129.6	B	43.1	48.2	53.4	B	34.5	37.2	43.3	<0.05
4-Hydroxyproline	A	80.8	95.8	121.4	B	17.9	20.8	27.0	B	17.8	19.7	28.3	<0.05
5-Oxoproline	A	80.9	104.6	116.8	B	27.6	30.5	38.9	B	28.6	32.0	36.2	<0.05
Alanine	A	90	98.7	110.6	B	16.4	18.9	21.5	B	15.9	18.0	19.4	<0.05
Arginine	A	70.2	99.0	130.3	B	16.3	18.4	25.0	B	13.3	17.6	23.7	<0.05
Ascorbic acid	A	84.4	95.2	118.0	B	45.6	50.7	51.8	B	44.1	51.7	59.0	<0.05
Citric acid	A	91.1	98.1	109.9	B	65.3	73.9	81.2	B	63.2	67.1	70.6	<0.05
Cystathionine	A	53.9	57.8	167.2	B	0.2	0.4	0.6	B	0.1	0.2	0.5	<0.05
Fumaric acid	A	78.8	96.7	122.8	B	28.4	35.7	45.2	B	35.8	44.0	49.5	<0.05
Galactose	A	80.8	99.9	119.3	B	43.2	55.8	70.1	B	52.3	53.9	62.4	<0.05
Glutamic acid	A	83.2	103.6	115	B	29.1	32.3	40.7	B	29.8	34.4	39.0	<0.05
Glutamine	A	74.8	98.3	126	B	17.2	21.9	24.2	B	15.4	15.5	20.1	<0.05
Glycine	A	78.4	98.6	122.3	B	14.9	17.4	21.9	B	17.3	27.8	31.3	<0.05
Homocysteine	A	82.6	91.0	121.9	B	3.3	4.2	6.7	B	1.8	2.6	7.3	<0.05
Homocystine	A	53.9	69.7	161.3	B	1.2	3.6	9.7	B	4.7	8.1	11.5	<0.05
Hypotaurine	A	73.5	85.6	133.7	B	20.4	25.9	34.9	B	20	24.6	28.9	<0.05
Inositol	A	91.4	98.9	109.1	B	39.5	46.5	56.6	B	38.8	42.9	49.2	<0.05
Isoleucine	A	85.0	98.1	115.9	B	44.4	49.7	51.4	B	41	44.5	54.8	<0.05
Lactose	A	42.8	109.5	152.5	B	8.3	10.7	16.5	B	9.9	12.8	16.0	<0.05

P7, P28, and P42; 7, 28, and 42 days of age, respectively. Q1, Q2, and Q3; lower quartile, median quartile, and upper quartile, respectively. KW, Kruskal-Wallis test; Different letters in the same line denote significantly different according to Steel-Dwass test ($p < 0.05$). The unit for metabolites is the relative value. n=5 in each group.

Table S2. (continued)

		P7				P28				P42			KW
		**Q1**	**Q2**	**Q3**		**Q1**	**Q2**	**Q3**		**Q1**	**Q2**	**Q3**	***p*-value**
Leucine	A	60.5	100.4	139.3	B	24.5	34.0	40.2	B	25.7	38.7	47.1	<0.05
Malic acid	A	76.3	90.5	128.5	B	31.5	33.1	49.0	B	34.5	42.9	51.7	<0.05
Malonic acid	A	77.2	98.1	123.8	B	12.8	24.8	46.4	B	12.5	31.7	35.7	<0.05
Maltose	A	50.3	85.9	156.7	B	9.4	10.4	14.6	B	7.0	9.8	12.5	<0.05
Mannitol	A	80.9	95.5	121.4	B	9.8	12.0	19.3	B	9.8	10.4	25.3	<0.05
Margaric acid	A	66.4	93.4	136.9	B	21.3	24.0	33.5	B	18.8	24.7	26.1	<0.05
Ornithine	A	75.7	94.0	127.3	B	17.8	21.2	27	B	15.2	20.2	24.2	<0.05
Phenylalanine	A	90.9	99.1	109.5	B	25.7	30.0	30.7	B	29.2	32.3	37	<0.05
Proline	A	86.5	95.4	115.8	B	8.4	14.8	19.8	B	8.6	9.9	12.3	<0.05
Putrescine	A	66.7	94.3	136.1	B	18.9	22.6	28	B	11.3	15.5	17.8	<0.05
Ribonic acid	A	24.6	103.5	173.6	B	1.2	1.6	5.5	B	0.1	1.4	3.7	<0.05
Sarcosine	A	69.4	96.7	132.3	B	9.4	13.4	29.9	B	8.0	10.3	11.3	<0.05
Serine	A	86.1	103.0	112.4	B	22.8	24.9	34.9	B	21.5	23.5	26.6	<0.05
Spermidine	A	70.7	110.8	123.9	B	30.8	37.3	38.8	B	14.7	24.6	27.3	<0.05
Threonic acid	A	50.6	60.0	169.4	B	8.9	9.2	14.1	B	8.7	12.1	13.9	<0.05
Threonine	A	91.8	102.3	107.1	B	25.4	26.7	32.6	B	20.4	24.0	25.0	<0.05
Tryptophan	A	88.1	95.1	114.3	B	32.3	33.9	45.7	B	34.0	35.1	44.6	<0.05
Valine	A	73.8	101.6	125.4	B	31.2	38.3	41.3	B	35.7	41.1	49.0	<0.05
Mesaconic acid	A	79.6	81.1	129.9	B	19.1	24.0	35.3	C	10.1	10.9	14.4	<0.05

P7, P28, and P42; 7, 28, and 42 days of age, respectively. Q1, Q2, and Q3; lower quartile, median quartile, and upper quartile, respectively. KW, Kruskal-Wallis test; Different letters in the same line denote significantly different according to Steel-Dwass test ($p < 0.05$). The unit for metabolites is the relative value. n=5 in each group.
