# Supplementary material for: Metabolism of Imidazole Dipeptides, Taurine, Branched-Chain Amino Acids, and Polyamines of the Breast Muscle Are Affected by Post-Hatch Development in Chickens
Source: Metabolites. 2022 Jan 17;12(1):86. doi: 10.3390/metabo12010086 (PMC8778354; doi:10.3390/metabo12010086)
Supplement: Supplementary file 1 [file metabolites-12-00086-s001.zip › Table S3 Metabolites increased in the breast muscle.pdf]

Table S3. Metabolites increased during postnatal development in the breast muscle of chickens

		P7				P28				P42			KW
		Q1	Q2	Q3		Q1	Q2	Q3		Q1	Q2	Q3	*p*-value
Glycerol 3-phosphate	B	0.0	63.3	218.4	A	884.4	1459.7	3994	A	811.7	992.3	3528	<0.05
Sedoheptulose 7-phosphate	B	49.0	92.7	154.6	AB	63.3	2247.1	14820.6	A	13008.7	21601.3	91442.2	<0.05
Uric acid	B	82.8	97.2	118.6	AB	128.4	141.0	192.2	A	146.8	162.7	232.6	<0.05

P7, P28, and P42; 7, 28, and 42 days of age, respectively. Q1, Q2, and Q3; lower quartile, median quartile, and upper quartile, respectively. KW, Kruskal-Wallis test; Different letters in the same line denote significantly different according to Steel-Dwass test ($p < 0.05$). The unit for metabolites is the relative value. n=5 in each group.
