# Supplementary material for: Metabolism of Imidazole Dipeptides, Taurine, Branched-Chain Amino Acids, and Polyamines of the Breast Muscle Are Affected by Post-Hatch Development in Chickens
Source: Metabolites. 2022 Jan 17;12(1):86. doi: 10.3390/metabo12010086 (PMC8778354; doi:10.3390/metabo12010086)
Supplement: Supplementary file 1 [file metabolites-12-00086-s001.zip › Table S4 Other metabolites in the breast muscle.pdf]

Table S4. Other metabolite levels during postnatal development in the breast muscle of chickens

		P7				P28				P42			KW
		**Q1**	**Q2**	**Q3**		**Q1**	**Q2**	**Q3**		**Q1**	**Q2**	**Q3**	***p*-value**
3-Hydroxypropionic acid	A	78.3	107.8	117.8	A	109.3	134.3	160	A	127.5	173.3	187.9	<0.05
Dimethylglycine	A	80.3	104.6	117.4	A	55.3	63.8	77.8	A	58.9	59.8	74.1	<0.05
Fructose 6-phosphate	A	50.6	77	160.9	A	19.2	36.8	96.2	A	6.2	10.4	37.8	<0.05
Glucose 6-phosphate	A	55.3	79.9	154.8	A	22.2	42.4	101.5	A	5.5	11.4	40.1	<0.05
Hydroxylamine	A	91.7	94.4	111.1	A	111.1	126.4	133.3	A	97.9	110.7	113.5	<0.05
Lactic acid	A	95.6	99.0	104.9	A	107.5	113.0	120.6	A	103.2	106.9	118.3	<0.05
N-Acetylaspartic acid	A	81.6	100.3	118.2	A	113.5	125.3	154.1	A	122.6	139.6	144.1	<0.05
O-Phosphoethanolamine	A	67.1	87.1	139.3	A	35.1	42.5	66.6	A	31.9	40.4	58.6	<0.05
Oxalacetic acid	A	85.0	105.7	112.2	A	119.1	133.1	136.2	A	106.6	109.6	119.6	<0.05
Tagatose	A	27.5	93.3	175.9	A	5.0	14.5	29.9	A	0.9	15.3	36.5	<0.05
2-Hydroxybutyric acid		68.6	108.6	127.1		95.3	187.6	212.9		147.6	178.7	240.8	0.055
Glucose		93.6	101.7	105.5		74.9	87.0	97.4		80.9	84.2	96.4	0.065
Pyruvic acid		86.4	104.4	111.4		35.3	75.2	93.2		59.1	67.1	89.7	0.067
Palmitic acid		97.0	98.8	103.6		105.7	114	139.9		101.4	114.3	120.9	0.085
Inosine		83.2	94.3	119.7		35.2	68.1	92.0		50.0	69.4	85.7	0.087
Sucrose		62.9	70.6	151.8		47.1	58.9	85.4		48.2	51.1	66.4	0.102
Galacturonic acid		82.8	101.3	116.5		91.3	118.2	123.1		113.5	128.3	149.4	0.114
Mannose 6-phosphate		50.1	78.8	160.5		34.4	63.0	101.1		7.1	23.2	56.2	0.114
Phosphoric acid		95.8	99.5	104.5		71.8	113.6	120.7		103.2	106.8	118.6	0.141
Succinic acid		75.1	105.6	122.1		93.1	155.9	194.6		59.3	83.8	119.3	0.141
Oleic acid		47.8	71.3	166.5		0.1	7.9	100		5.5	8.1	66.0	0.145
Adenosine		71.4	106.8	125.2		45.1	138.3	227.2		106.8	313.1	441.8	0.151
Asparagine		61.4	79.1	149.0		34.5	64.7	87.4		26.6	46.0	76.6	0.151
Oxalic acid		72.1	102.4	126.7		40.5	54.3	213.5		30.6	42.5	84.1	0.151
Glycerol		81.6	98.7	119.0		27.9	62.4	97.1		69.6	97.9	110.5	0.179
Ribose 5-phosphate		10.5	66.6	206.2		58.4	71.5	100.4		94.3	102.5	125.2	0.185
Cystamine		75.5	95.2	126.9		111.1	153.7	169.8		98.6	121.6	147.9	0.208
Boric acid		84.5	89.2	121		106.3	115.4	129.3		87.5	92.4	119.7	0.221
Ethylhydracrylic acid		57.9	97.7	143.2		31.0	105.4	219.4		112.5	258.5	279.9	0.221

P7, P28, and P42; 7, 28, and 42 days of age, respectively. Q1, Q2, and Q3; lower quartile, median quartile, and upper quartile, respectively. KW, Kruskal-Wallis test; Different letters in the same line denote significantly different according to Steel-Dwass test ($p < 0.05$). The unit for metabolites is the relative value. n=5 in each group.

Table S4. (continued)

	P7			P28			P42			KW
	**Q1**	**Q2**	**Q3**	**Q1**	**Q2**	**Q3**	**Q1**	**Q2**	**Q3**	***p*-value**
Gluconic acid	4.8	114.6	187.8	27.5	33.2	49.2	1.2	8.9	36.5	0.247
5'-Methylthioadenosine	34.5	51.1	189.9	29.9	814.6	1157.5	178	550.0	1907.4	0.275
Aspartic acid	89.5	99.4	110.8	81.8	93.5	100.7	89.2	90.4	92.4	0.31
Stearic acid	96.1	100.1	103.8	102.1	105.5	114.2	96.6	109.0	117.3	0.326
5-Methoxytryptamine	43.6	104.6	154	54.9	113.7	191.0	40.0	66.5	106.8	0.403
Citraconic acid	59.0	66.2	157.9	42.5	129.0	400.5	99.9	109.8	143.5	0.403
Monostearin	89.3	105.3	108.1	88.4	107.0	123.7	103	107.1	136.2	0.403
Urea	76.9	95.7	125.2	29.2	90.1	105.2	65.4	68.9	92.2	0.403
Adenine	70.4	107.3	126	80.5	90.8	97.3	90.3	96.6	103.1	0.468
3-Aminopropanoic acid	58.3	104.7	139.3	98.7	121.8	169.7	63.8	98.8	148.7	0.472
Uridine	92.0	101.2	107.4	94.7	111.3	119.3	99.5	103.7	113.5	0.472
Uracil	84.9	102.6	113.8	92.6	113.9	126.3	93.1	100.9	103.3	0.527
Creatinine	66.3	99.4	134	79.3	111.3	154.1	92.9	122.0	145.9	0.533
2-Ketoadipic acid	33.7	109.6	161.5	3.9	30.1	129.2	3.8	23.8	132.7	0.543
Glycolic acid	70.8	102.9	127.8	98.2	109.3	120.5	101.2	111.8	149	0.613
2-Methyl-3-hydroxybutyric acid	11.2	118.6	179.5	27.7	126.0	255.4	48.9	72.0	112.1	0.651
Nicotinamide	67.9	100.1	132.0	78.5	123.8	131.5	98.0	104.7	151.6	0.677
Inosine monophosphate	82.3	87.0	124.2	82.2	107.4	1624.7	0.3	63.5	5211.1	0.691
3-Phosphoglyceric acid	84.5	90.3	120.4	70.5	89.3	800.4	47.3	81.3	557.4	0.733
Hypoxanthine	40.1	133.0	143.4	109.8	128.1	143.6	103.7	126.1	128.7	0.756
2-Aminoadipic acid	82.5	96.0	119.5	84.3	99.6	129.8	91.7	100.2	128.4	0.852
2-Keto-isovaleric acid	48.1	78.9	162.4	62.0	87.5	146.9	64.7	99.7	133.7	0.852
Arabitol	52.6	105.9	144.5	75.6	79.9	103	67.4	83.0	99.6	0.887

P7, P28, and P42; 7, 28, and 42 days of age, respectively. Q1, Q2, and Q3; lower quartile, median quartile, and upper quartile, respectively. KW, Kruskal-Wallis test; Different letters in the same line denote significantly different according to Steel-Dwass test ($p < 0.05$). The unit for metabolites is the relative value. n=5 in each group.
