# Supplementary material for: Metabolism of Imidazole Dipeptides, Taurine, Branched-Chain Amino Acids, and Polyamines of the Breast Muscle Are Affected by Post-Hatch Development in Chickens
Source: Metabolites. 2022 Jan 17;12(1):86. doi: 10.3390/metabo12010086 (PMC8778354; doi:10.3390/metabo12010086)
Supplement: Supplementary file 1 [file metabolites-12-00086-s001.zip › Table S5 Metabolites decreased in the plasma.pdf]

Table S5. Metabolites decreased during postnatal development in the plasma of chickens

		P7				P28				P42			KW
		Q1	Q2	Q3		Q1	Q2	Q3		Q1	Q2	Q3	*p*-value
meso-Erythritol	A	90.4	103.8	107.8	A	72.4	83.5	86.1	B	45	51.3	59.4	<0.05
2-Hydroxy-3-methylvaleric acid	A	51.6	88.6	154.1	AB	20.0	41.4	62.6	B	8.7	9.9	14.3	<0.05
Ribonic acid	A	53.8	96.0	148.2	AB	6.0	10.1	34.1	B	1.1	3.7	30.9	<0.05
2-Ketoisocaproic acid	A	86.0	96.6	115.7	B	58.3	70.6	75.0	AB	48.8	58.9	78.4	<0.05
Gluconic acid	A	86.8	101.0	112.7	B	30.7	34.3	42.8	AB	45.6	51.1	71.3	<0.05
Inositol	A	94.2	98.0	106.8	B	124.4	141.7	145.7	AB	88.2	112.1	145.6	<0.05
2-Deoxytetronic acid	A	72.4	94.4	130.4	B	1.1	2.5	14.6	B	0.0	0.7	19.9	<0.05
2-Hydroxyisovaleric acid	A	74.2	78.2	136.6	B	28.3	37.4	54.0	B	19.2	21.6	27.0	<0.05
2-Keto-isovaleric acid	A	71.9	97.0	129.6	B	21.9	30.2	50.0	B	16	27.1	48.9	<0.05
3-Methyl-2-oxovaleric acid	A	82.8	94.8	119.8	B	41.5	54.7	65.0	B	34.8	47	61.5	<0.05
Dimethylglycine	A	88.0	101.1	111.5	B	154.1	156.0	202.2	B	145.1	159.7	191	<0.05
Galacturonic acid	A	83.0	98.6	117.7	B	20.3	33.2	45.3	B	8.6	23.6	39.1	<0.05
Glucaric acid	A	85.7	90.0	119.3	B	42.2	48.3	58.1	B	24.5	34.6	51.2	<0.05
Lactitol	A	70.3	107.5	125.9	B	16.5	27.3	34.6	B	17.1	19.9	26.6	<0.05
Maltose	A	69.2	83.6	139.0	B	28	60.3	65.0	B	31.8	52.1	58.3	<0.05
Mannitol	A	76.2	99.4	124.1	B	11.9	19.2	28.8	B	5.8	9.4	17.8	<0.05
Phenylalanine	A	90.5	101.9	108.5	B	49.5	50.4	68.8	B	58.6	82.7	86.7	<0.05
Sucrose	A	67.9	83.5	140.3	B	19.2	27.0	42	B	14.5	16.1	33.4	<0.05
3-Hydroxyisovaleric acid	A	54.9	111.1	139.6	B	19.6	21.6	25.6	C	15.3	17.0	17.4	<0.05
Homocystine	A	74.4	109.5	120.8	B	15.5	27.2	31.5	C	4.9	6.3	7.9	<0.05
Threitol	AB	87.7	100.9	111.8	A	95.1	95.6	104.0	B	55.7	65.2	80.2	<0.05

P7, P28, and P42; 7, 28, and 42 days of age, respectively. Q1, Q2, and Q3; lower quartile, median quartile, and upper quartile, respectively. KW, Kruskal-Wallis test; Different letters in the same line denote significantly different according to Steel-Dwass test ($p < 0.05$). The unit for metabolites is the relative value. n=5 in each group.
