# Supplementary material for: Metabolism of Imidazole Dipeptides, Taurine, Branched-Chain Amino Acids, and Polyamines of the Breast Muscle Are Affected by Post-Hatch Development in Chickens
Source: Metabolites. 2022 Jan 17;12(1):86. doi: 10.3390/metabo12010086 (PMC8778354; doi:10.3390/metabo12010086)
Supplement: Supplementary file 1 [file metabolites-12-00086-s001.zip › Table S6 Metabolites increased in the plasma.pdf]

Table S6. Metabolites increased during postnatal development in the plasma of chickens

		P7				P28				P42			KW
		Q1	Q2	Q3		Q1	Q2	Q3		Q1	Q2	Q3	*p*-value
Creatinine	B	48.3	91.6	155.8	A	652.5	691.7	785.2	A	736.2	849.4	1151.3	<0.05
Glucose 6-phosphate	B	75.7	97.8	125.4	A	573.0	845.9	1166.4	A	759.7	913.7	1171.0	<0.05
Glutamic acid	B	87.4	102.7	111.3	A	155.0	232.2	244.5	A	188.5	228.9	291.6	<0.05
Glyceric acid	B	85.9	96.0	116.0	A	138.2	145.4	162.1	A	142.6	157.8	183.7	<0.05
Glycerol 3-phosphate	B	85.8	90.5	118.9	A	218.9	271.3	344.7	A	206.8	235.9	307.8	<0.05
Inosine	B	53.8	102.7	144.8	A	239.8	475.4	639.3	A	318.9	582.4	719.5	<0.05
Norepinephrine	B	37.8	93.3	165.6	A	942.3	1731.7	3291.4	A	1506.4	1761.4	2176.2	<0.05
O-Phosphoethanolamine	B	75.8	101.4	123.5	A	361.4	507.0	580.9	A	363.2	400.9	507.4	<0.05
Phosphoric acid	B	80.6	96.5	121.2	A	156.0	172.2	191.6	A	160.6	167.5	205.2	<0.05
Pyruvic acid	B	83.1	102.4	115.7	A	168.4	178.9	208.5	A	146.7	157.1	192.7	<0.05
Succinic acid	B	60.0	81.1	149.5	A	413.7	1073.6	2542.0	A	1230.6	2329.9	5170.9	<0.05
2-Aminoethanol	B	92.6	103.1	105.9	A	117.5	124.4	145.0	AB	80.4	114.6	124.7	<0.05
Fucose	B	89.9	92.9	113.6	A	121.3	126.4	140.9	AB	107.5	119.0	134.0	<0.05
Tricarballylic acid	B	66.3	107.0	130.2	A	193.1	260.5	718.8	AB	136.5	170.7	422.2	<0.05
Fumaric acid	B	91.9	102.6	106.8	AB	108.7	139.7	196.2	A	161.2	278.2	352.7	<0.05
Guanosine	B	46.6	89.7	158.5	AB	106.0	261.0	294.7	A	220.6	265.8	361.4	<0.05
Hypoxanthine	B	70.1	78.0	140.9	AB	203.0	322.2	368.7	A	227.5	343.1	371.9	<0.05
Psicose	AB	74.9	95.9	127.1	B	66.4	79.4	89.6	A	146.5	176.0	259.5	<0.05

P7, P28, and P42; 7, 28, and 42 days of age, respectively. Q1, Q2, and Q3; lower quartile, median quartile, and upper quartile, respectively. KW, Kruskal-Wallis test; Different letters in the same line denote significantly different according to Steel-Dwass test ($p < 0.05$). The unit for metabolites is the relative value. n=5 in each group.
