# Supplementary material for: Metabolism of Imidazole Dipeptides, Taurine, Branched-Chain Amino Acids, and Polyamines of the Breast Muscle Are Affected by Post-Hatch Development in Chickens
Source: Metabolites. 2022 Jan 17;12(1):86. doi: 10.3390/metabo12010086 (PMC8778354; doi:10.3390/metabo12010086)
Supplement: Supplementary file 1 [file metabolites-12-00086-s001.zip › Table S7 Other metabolites in the plasma.pdf]

Table S7. Other metabolite levels during postnatal development in the plasma of chickens

		P7				P28				P42			KW
		Q1	Q2	Q3		Q1	Q2	Q3		Q1	Q2	Q3	*p*-value
2-Methyl-3-hydroxybutyric acid	A	80.5	98.4	120.3	A	49.7	56.2	78.7	A	52.7	68.7	86.6	<0.05
5-Oxoproline	A	90.6	100	109.4	A	113.9	121	142.9	A	104.3	115.5	147.5	<0.05
Aconitic acid	A	90.8	97.1	110.6	A	54.6	69.4	86.8	A	54.8	78.2	88.0	<0.05
Cystine	A	86.5	100.8	113.1	A	38.2	55.1	75.9	A	60.2	94.6	103.3	<0.05
Glucuronic acid	A	88.3	95.2	114.1	A	90.9	98.0	103.5	A	44.6	79.4	88.6	<0.05
Glycerol	A	94.6	97.5	106.7	A	105.3	115.9	118.2	A	112.4	125.9	141.6	<0.05
Mannose	A	80.3	96.7	121.3	A	51.7	66.8	78	A	26.5	27.8	58.9	<0.05
Threonic acid	A	87.8	100.9	111.7	A	77.4	79.7	84.7	A	61.5	67.5	82.2	<0.05
Ureidopropionic acid	A	89.6	99.2	110.8	A	130.6	146.4	163.5	A	90.4	127.6	142.8	<0.05
Valine	A	79.4	110.0	115.6	A	48.8	54.2	77.3	A	64.3	81.2	83.0	<0.05
Xylitol	A	84.8	95.5	117.4	A	56	68.4	78.5	A	71.8	98.8	103.5	<0.05
Malic acid		88.2	93.2	115.2		98.2	147.3	202.6		129.3	252.4	296.2	0.056
Uric acid		95.9	100.7	103.8		75.7	87.4	91.6		76.6	83.1	99.8	0.059
Leucine		83.3	103.5	114.9		56.9	62.1	85.5		75.3	88.3	94.4	0.061
Proline		90.5	99.8	109.6		50.1	76.0	88		64.7	77.2	101.8	0.061
Tryptophan		86.3	97.9	114.7		95.5	100.7	129.4		112.3	127.3	144.8	0.061
Cystathionine		78.5	110.8	116.1		52.1	59.4	90.6		39	47.0	70.8	0.075
Methylmalonic acid		78.5	105.7	118.6		71.3	74.0	90.3		50.5	63.5	90.5	0.075
Xanthine		75.5	96.7	126.2		120.4	144.0	173.2		101.9	200.4	251.3	0.081
2-Aminobutyric acid		81.4	90.5	123.4		102.8	110.3	120.5		72.8	78.7	100.1	0.085
Tyrosine		87.7	95.0	114.8		90.3	114.7	138.3		117.1	138.7	153.9	0.085
Uracil		85.9	96.4	115.9		113.8	120.1	140.8		102.2	116.0	148.0	0.087
Glutamine		90.5	95.9	111.6		109.8	119.8	130.4		100.1	124.0	139.7	0.088
Ribose		87.4	102.4	111.4		110.9	119.4	126.7		80.4	101.9	125.8	0.088
Xylose		76.4	105.7	120.8		82.9	91.3	95.3		63.3	69.5	82.1	0.090
2-Ketoglutaric acid		85.1	102.4	113.6		108.4	126.6	137.8		99.1	115.3	127.4	0.105
Sarcosine		79.2	86.9	127.4		105.5	156.9	237.3		102.7	113.4	214.4	0.108
Tagatose		17.0	37.7	214.2		139.9	582.2	960.6		21.9	56.4	335.1	0.108
Hypotaurine		64.7	84.1	143.3		53.6	55.7	82.3		78.9	109.9	113.3	0.112

P7, P28, and P42; 7, 28, and 42 days of age, respectively. Q1, Q2, and Q3; lower quartile, median quartile, and upper quartile, respectively. KW, Kruskal-Wallis test; Different letters in the same line denote significantly different according to Steel-Dwass test ($p < 0.05$). The unit for metabolites is the relative value. n=5 in each group.

Table S7. (continued)

	P7			P28			P42			KW
	Q1	Q2	Q3	Q1	Q2	Q3	Q1	Q2	Q3	*p*-value
Malonic acid	79.3	94.9	123.2	60.7	72.5	85.7	69.1	74.2	97.4	0.125
3-Aminoisobutyric acid	86.9	89.9	118.1	68.1	75.9	99.5	57.9	59.4	92.1	0.137
Acetoacetic acid	87.3	97.5	113.9	96.7	103.7	116.4	103	121.5	134.8	0.137
Arabinose	75.0	86.1	132.0	66.6	70.0	121.1	57.6	66.3	78.2	0.141
Lactic acid	91.3	101.3	108.1	105.9	110.6	112	98.3	101.1	115.7	0.141
Methionine	77.7	97.7	123.4	77.8	86.7	99.8	95.8	117.8	138.6	0.141
Urea	83.9	90.3	121.0	76.6	80.0	89.4	67.1	82.6	94.9	0.145
Arabitol	72.8	101.2	126.6	119.3	124.3	142.9	104.2	115.2	154.6	0.151
4-Hydroxyproline	81.0	88.7	124.7	62.9	65.7	83.9	64.0	67.9	125.6	0.160
Isoleucine	87.2	95.5	115.1	65.5	70.7	92.4	70.7	85.3	95.1	0.164
Benzoic acid	73	83.2	135.4	125	143.5	146.5	81.4	101.1	143.7	0.179
Alanine	91.0	100.6	108.7	80.5	92.1	98.1	72.0	83.6	103.5	0.196
Elaidic acid	66.2	95.0	136.3	56.9	71.0	90.9	25.3	65.1	89.8	0.196
Threonine	89.6	96.9	112.0	80.4	91.5	101.6	74.6	85.9	98.8	0.208
Monostearin	93.1	104	104.9	59.3	92.2	96.8	81.2	89.4	114.5	0.230
Palmitic acid	91.4	104.3	106.4	89.4	92.7	98.2	68.9	87.6	111.5	0.230
Galactose	0.0	51.7	224.1	34.2	100.4	6017.7	61.7	227.0	2867.9	0.28
Mannose 6-phosphate	34.8	63.2	183.6	50.4	139.2	307.4	82.6	92.2	281.7	0.281
Margaric acid	88.8	100.5	110.9	93.3	97.5	102.3	61.8	86.3	111.9	0.281
3-Hydroxybutyric acid	73.3	92.9	130.3	70.1	86.4	96.4	91.1	98.6	125.1	0.289
Allose	77.1	95.6	125.1	107.4	123.2	206.7	90	110.0	214.4	0.289
Uridine	83.9	105.2	113.5	108.5	112.8	158.9	98.1	109.2	200.3	0.310
Hydroxylamine	84.3	104.4	113.5	77.8	83.5	99.3	70.1	81.2	99	0.326
Inositol phosphate	72.1	83.2	136.3	44.7	49.5	98.8	58.6	70.4	82.2	0.336
Glucose	90.2	98.2	110.7	71.4	90.5	98.7	54.4	93.4	106.1	0.357
Nonanoic acid	81.9	105.4	115.4	96.8	115.2	126.6	91.2	130.2	141.9	0.357
Serine	82.1	97.7	119	105.2	118.2	121.9	92.9	108.0	130.2	0.357
Stearic acid	94.8	101.7	104.3	93.1	101.5	102.3	80.2	91.8	111.8	0.379
15-Anhydro-glucitol	64.6	79.0	145.9	32.7	47.9	128	32.8	54.1	101.7	0.395

P7, P28, and P42; 7, 28, and 42 days of age, respectively. Q1, Q2, and Q3; lower quartile, median quartile, and upper quartile, respectively. KW, Kruskal-Wallis test; Different letters in the same line denote significantly different according to Steel-Dwass test ($p < 0.05$). The unit for metabolites is the relative value. n=5 in each group.

Table S7. (continued)

	P7			P28			P42			KW
	Q1	Q2	Q3	Q1	Q2	Q3	Q1	Q2	Q3	*p*-value
Citric acid	90.5	101.9	108.6	99.8	111.6	120.6	88.8	96.4	117.2	0.395
Fructose 6-phosphate	1.6	87.8	204.5	43.6	104.5	446.1	10.1	24.3	117.0	0.395
3-Hydroxypropionic acid	91.9	100.1	108	97.0	101.5	103.6	89.9	93.5	106.2	0.403
Glycolic acid	90.1	96.6	111.6	92.0	107.3	115.1	76.4	84.6	111.5	0.403
Glycine	87.5	92.7	116.2	77.5	91.0	107.3	87.7	109.2	132.3	0.445
2-Hydroxyisobutyric acid	62.9	105.3	134.5	68.6	81.7	93.5	66.1	76.8	96.5	0.454
Dopamine	84.4	94.9	118.1	99.7	103.9	119.3	82.9	94.2	128.4	0.454
Linoleic acid	65.2	105.0	132.3	62.2	70.7	107.2	53.3	74.8	101.4	0.454
Norvaline	8.8	14.0	234.2	39.9	121.2	228.4	76.9	196.4	264.5	0.482
Isomaltose	58.7	115.2	133.7	74.3	141.8	165.2	58.6	109	169.1	0.512
Boric acid	85.2	91.8	118.9	92.5	100.8	111.9	72.4	87.0	113.9	0.527
Adenosine	78.9	109.0	116.6	68.6	105.6	144.6	85.6	118.3	151.9	0.533
Ascorbic acid	28.6	118.5	162.2	80.2	93.2	458.0	1.1	9.6	462.2	0.566
Homocysteine	69.0	110.2	125.9	68.6	79.5	122.8	51.0	68.6	110.5	0.566
Dihydroorotic acid	49.9	92.6	153.8	49.8	75.7	98.5	54.0	61.9	85.1	0.595
5-Methoxytryptamine	33.4	92.3	170.5	67.5	139.1	217.8	64.2	98.6	326.5	0.613
Lysine	72.4	111.8	121.8	92.8	101.0	114.2	63.5	102.7	107.0	0.613
Myristic acid	88.6	99.9	111.4	93.9	94.1	100.0	68.1	91.3	114.7	0.613
Ornithine	88.7	100.4	111.1	78.4	101.3	124.8	93.8	109.5	133.9	0.619
N-Acetylmannosamine	81.7	104.2	116.2	96.1	106.3	124.9	42.2	89.8	129.4	0.691
Asparagine	75.9	103.3	122.4	62.6	80.5	124.5	69.6	106.4	140.0	0.756
Lactose	70.0	117.3	121.4	75.3	105.1	131.0	44.3	94.4	124.7	0.779
3-Phenyllactic acid	72.9	105.0	124.6	102.6	107.5	112.3	86.4	104.8	133.3	0.811
4-Aminobutyric acid	89.6	105.3	107.7	98.8	103.0	105.9	93.5	96.9	106.7	0.811
Sorbose	77.2	101.1	122.3	91.1	102.6	114.3	50.3	78.0	128.0	0.811
Cysteine	62.0	105.4	135.3	98.9	115.3	143.9	92.7	106.4	148.7	0.827
2-Hydroxybutyric acid	85.5	93.0	118.0	87.2	96.0	113.5	81.2	108.0	120.5	0.990
Cystamine	71.4	99.7	128.8	81.5	100.9	133.8	85.3	101.3	109.1	0.990

P7, P28, and P42; 7, 28, and 42 days of age, respectively. Q1, Q2, and Q3; lower quartile, median quartile, and upper quartile, respectively. KW, Kruskal-Wallis test; Different letters in the same line denote significantly different according to Steel-Dwass test ($p < 0.05$). The unit for metabolites is the relative value. n=5 in each group.
