# Supplementary material for: Metabolism of Imidazole Dipeptides, Taurine, Branched-Chain Amino Acids, and Polyamines of the Breast Muscle Are Affected by Post-Hatch Development in Chickens
Source: Metabolites. 2022 Jan 17;12(1):86. doi: 10.3390/metabo12010086 (PMC8778354; doi:10.3390/metabo12010086)
Supplement: Supplementary file 1 [file metabolites-12-00086-s001.zip › Table S8 LC-MSMS conditions.pdf]

Table S8. Retention time and instrument settings for the analyzed compounds.

Compound name	Retention time (min)	Q1 (m/z)	Q2 (m/z)	Dwell (s)	Cone voltage (V)	Collision energy (V)
Histidine ring ($^{13}C_1$)	1.7	327.3	171.0	0.128	24	21
Taurine	2.3	296.1	171.0	0.061	18	15
Glutamine (d_5)	2.8	322.2	171.0	0.061	22	24
Carnosine	2.8	397.2	171.0	0.061	24	21
Anserine	3.1	411.3	171.0	0.128	24	21
Balenine	3.3	411.3	171.0	0.128	24	21
