# Supplementary material for: Metabolism of Imidazole Dipeptides, Taurine, Branched-Chain Amino Acids, and Polyamines of the Breast Muscle Are Affected by Post-Hatch Development in Chickens
Source: Metabolites. 2022 Jan 17;12(1):86. doi: 10.3390/metabo12010086 (PMC8778354; doi:10.3390/metabo12010086)
Supplement: Supplementary file 1 [file metabolites-12-00086-s001.zip › Table S9 Primers for PCR.pdf]

Table S9. Nucleotide sequence of primers used in RT-qPCR analyses

Gene	GeneBank accession number	Primer sequence (5′→3′)	Product size (bp)
CNDP1	XM_004935199.3	GCTGGAAAACTGACCCTTTCAC	138bp
		CTGGCATAGCTCTTTTCAAGGC	
CARNMT1	KR998071.1	TGCTTGCCAAGGAAATGAATGG	116bp
		TGCTAAACTGATGAATCCAGGGA	
CARNS	NM_001172593.1	GCCCACTGGATATGACTGTGTG	100bp
		CACAGCACCATGGCACCT	
CTH	XM_422542.5	AGCCATTCCGTCTCCCTTTG	111bp
		TCAAGGAATCGACCCACAGC	
BCKDH E1a	AB035083.1	ACCTCTTCTCCGATGTGTACCG	112bp
		TCGTAGAGCTCCATGGGGTAAT	
BCKDH E1b	AB035084.1	TTGGATTTGGTATTGGAGTTGCT	125bp
		AGAGCGGTAACGATACTTTGCTG	
BCAT1	XM_004937974.1	CAGCAGGTTCTGTGGCTATACG	115bp
		AAGGTGGAGTTGCCAGTTCATT	
ODC	NM_001167766.1	GCCAGAGTTGCAAGTTGGTG	129bp
		TGCCATGCTGGTCTTGACAT	
AMD	NM_001012569.1	CCACCCTCTTACTGCAAGCA	124bp
		TACTCCTGGTGGGAAGGCTT	
INSR	XM_001233398.5	GTCTGCTTTTCTCCCCTCCACA	146bp
		GACAACCAGTCAACTTGGCAAA	
IGF1R	NM_205032.1	GGCCATACGGATTGAGAAGAAC	110bp
		TCGGAGGCTTATTTCCAACAAT	
IRS1	XM_004951101.1	GCATTCAGGAAACGGACTCACT	120bp
		GGGGTAAGAAGGCAACATCTCC	
IRS2	XM_425588.4	ACTCGGACAGCTTCTTCTTCAT	152bp
		GAACTCGGACAGCTCCTTTAGA	
RPS17	NM_204217.1	AAGCTGCAGGAGGAGGAGAGG	136bp
		GGTTGGACAGGCTGCCGAAGT	

Abbreviations: *CNDP1*: carnosine dipeptidase 1, *CARNMT*: carnosine N-methyltransferase, *CARNS*: carnosine synthase, *CTH*: cystathionine gamma-lyase, *BCKDH E1a*: branched-chain alpha-keto acid dehydrogenase E1-alpha subunit, *BCKDH E1b*: branched-chain alpha-keto acid dehydrogenase E1-beta subunit, *BCAT1*: branched chain amino-acid transaminase 1, *ODC*: ornithine decarboxylase, *AMD*: adenosylmethionine decarboxylase, *INSR*: insulin receptor, *IGF1R*: insulin-like growth factor 1 receptor, *IRS1*: insulin receptor substrate 1, *IRS2*: insulin receptor substrate 2, *RPS17*: ribosomal protein S17
